# In-hospital hypothermia

**DOI:** 10.1186/cc11263

**Published:** 2012-06-07

**Authors:** Christian Storm

**Affiliations:** 1 Department of Nephrology and Medical Intensive Care Medicine, Charité Universitätsmedizin Berlin, Campus Virchow-Klinikum, Berlin, Germany

## Introduction

Mild therapeutic hypothermia after cardiac arrest has become standard in post-resuscitation care in many hospitals as it is recommended by current guidelines. The last update of guidelines by the European Resuscitation Council on post-cardiac arrest treatment in 2010 recommends hypothermia for every patient after cardiac arrest who remains unconscious after cardiac arrest [1]. In addition to milestone trials [2-4], current published retrospective data from the large Finnish registry showed in a large group of patients a significant reduction of hospital mortality of survivors of out-of-hospital cardiac arrest after implementation of hypothermia [5].

The mild therapeutic hypothermia procedure after cardiac arrest can be divided into three phases: introduction, maintenance and rewarming. The cooling techniques and devices to induce cooling of the cardiac arrest survivor can be separated into three main groups: conventional cooling (no device), non-invasive (surface) systems, and invasive (intravascular) systems (Table 1).

**Table 1:** 

Company	Device	Type of cooling	Cooling rate (°C/hour)	Auto feedback	Reusable
Philips	InnerCool RTx	Catheter	4.0 to 5.0	Yes	No
Zoll	Thermogard XP	Catheter	2.0 to 3.0	Yes	No
C.R. Bard	ArcticSun 5000	Surface adhesive pads	1.2 to 2.0	Yes	No
CSZ	Blanketrol III	Surface blanket	1.5	Yes	Yes
EMCOOLS	FLEX.PAD	Surface adhesive pads	3.5	No	No
MTRE	CritiCool	Surface blanket	1.5	Yes	no

The table gives the most common cooling devices with no claim to be complete. Cooling rates provided either by the company or at the company's Internet homepage. CSZ, Cincinnati Sub-Zero; MTRE, Medical ThermoRegulation Expertise.

## Cooling techniques

### Conventional cooling methods

The easiest way to induce hypothermia after cardiac arrest is by using cold saline (for example, 0.9% NaCl solution), crushed ice or ice bags. Kim and colleagues reported the safety and efficacy of the administration of up to 2 litres of 4°C cold saline to the patient after hospital admission [6]. Others published data using 30 ml/kg body weight of saline 0.9% NaCl or Ringer's lactate combined sometimes with ice bags, which led to an acceptable reduction of the temperature [7-10]. Furthermore cold saline as well as other methods like cooling caps and helmets have been evaluated for induction mainly in the preclinical setting [4,11,12]. Kliegel and colleagues pointed out that cold infusion alone is effective for induction but not for tight maintenance of the target temperature [13]. However, in at least one trial the combination of cold saline and ice packs was proven to be effective even to maintain temperature [7]. Focusing on the induction in the in-hospital setting, most authors rank cold saline and crushed ice more as effective adjuvant methods to be combined with a computer-controlled cooling device [10]. The big advantage of cold saline is its availability at almost every place in the hospital if provided and the low costs. Following the data available concerning different amounts of saline administered to the patient, a median amount of 1 to 2 litres of saline seems safe after cardiac arrest. To maintain target temperature with cold saline and ice bags seems to require a high binding of personnel method without a very precise influence on the central body temperature.

### Surface cooling methods

Surface, non-invasive devices have to be distinguished from intravascular, invasive devices. The range of available computer-controlled surface devices with automatic temperature feedback stretches from cooling blankets to be placed around the patient (Blanketrol III, Cincinnati Sub-Zero; CritiCool, Medical ThermoRegulation Expertise) to adhesive cooling pads (Arctic Sun, Bard). Heard and colleagues compared the adhesive Arctic Sun surface-cooling system with normal cooling blankets combined with ice bags. Although the reached target temperature within 4 hours was not significantly different between the groups, the Arctic Sun system cooled more rapidly down to the target temperature [14]. A current investigation from Norway compared the Arctic Sun surface (C.R. Bard) system (*n *= 92) with the invasive intravascular Coolgard (Alsius) system (*n *= 75) in cardiac arrest survivors. The authors concluded no significant differences concerning neurological outcome and survival at discharge. A limitation for interpretation of the device efficacy (cooling rate/hour) is the additional induction of cooling with cold saline and ice bags already in the emergency room [15]. A published case report described a severe skin peeling during hypothermia with the Arctic Sun system without a known history of skin problems or steroid therapy but with end-stage renal disease and coronary artery disease. This is the first severe adverse skin event towards the hydrogel pads known and the authors conclude that these skin lesions are very unusual as to be caused by the adhesive pads because exfoliative dermatitis is a rare syndrome and is often drug induced [16]. Thus adverse skin reactions should not normally be expected using this method of cooling.

Another surface feedback system using blankets is the CritiCool Pro system by Medical ThermoRegulation Expertise (MTRE, Israel). The patient is wrapped into the body-shaped heat exchange garment resulting in a median cooling rate of 0.7 ± 0.37°C/hour in a study by Laish-Farkash and colleagues [17]. The Cincinnati Sub-Zero system has been compared with the ArcticSun2000 (C.R. Bard) system by Mayer and colleagues for fever control in neurocritical care patients. The authors conclude the ArcticSun system to be superior to the Cincinnati Sub-Zero system due to the maintenance of normothermia, a higher cooling rate and better fever reduction, although shivering occurred more frequently in the ArcticSun group [18]. A surface cooling system without computer control and automatic temperature feedback is the EMCOOLS cooling system. The adhesive pads use a novel carbon cooling gel that has a high thermal conductivity resulting in a cooling rate of more than 3.5°C/hour. The Flex Pad needs to be adapted to the body size and shape. The feasibility trial of out-of-hospital surface cooling after return of spontaneous circulation (ROSC) in 15 survivors using the EMCOOLS system revealed a high median cooling rate of 3.3°C/hour, the target temperature of 33°C was reached approximately within 70 minutes (55 to 106 minutes) after the start of cooling and no skin lesions were observed [19]. A further novel system is the Life Recovery ThermoSuit system, which was developed mainly for fast induction of hypothermia by cold water (2°C) immersion due to a lack of a temperature feedback mechanism. The water circulates continuously directly on the patient's skin with a median cooling rate of 3°C/hour [20]. Published data by Howes and colleagues report the safe use of the ThermoSuit system in 24 cardiac arrest survivors reaching the target temperature(<34°C) within 37 minutes (range 14 to 81 minutes) [21]. After the patients have reached the target temperature, they have to be removed from the suit and cooling maintained with other methods.

### Endovascular cooling

Intravascular closed-loop cooling systems are also computer controlled with a temperature feedback. The Thermogard XP Temperature Management System (Zoll) provides both a central venous catheter with an additional closed loop balloon system with circulating water for cooling. The InnerCool RTx device (Philips) using the Accutrol catheter has a special feature with an integrated temperature sensor but no additional central intravascular access. A possible advantage of taking the temperature directly in the bloodstream is the avoidance of lag in core temperature measurement inherent in rectal and bladder sensors. The very precise temperature control is needed, taking the high average cooling rates of 4.0 to 5.0°C/hour into consideration. This cooling system will be under evaluation in the Rapid Endovascular Catheter Core Cooling combined with cold saline as an Adjunct to Percutaneous Coronary Intervention for the Treatment of Acute Myocardial Infarction (CHILL-MI) study. This study was started in 2012 to further investigate the safety and effectiveness of the endovascular cooling system in patients suffering from ST-elevation myocardial infarction (STEMI) and to confirm the data from the Rapid-MI-ICE trial [22]. In a subanalysis of the European Hypothermia After Cardiac Arrest trial (HACA), Holzer and colleagues retrospectively reviewed the efficacy and safety of the intravascular catheter system (Cool Gard 3000, Alsius) in 56 patients, revealing a cooling rate of 1.2°C/hour (IQR 0.7 to 1.5) without significant differences to other techniques concerning adverse events [23]. A study by Gillies and colleagues reported a good temperature control with endovascular cooling compared with conventional ice surface cooling [24]. After induction of cooling with cold saline, one group was continued to be cooled with ice (*n *= 41) whereas the other group was cooled with the Coolgard device (*n *= 42; Alsius). In summary, catheter cooling provided a more precise temperature control, better control during rewarming, less overcooling and failure to reach target temperature. Despite these advantages there was no difference concerning outcome between both relatively small groups [24]. The duration of time an intravascular catheter can be used as central intravenous access after rewarming is not well investigated so far. Al-Senani and colleagues evaluated the safety of the Icy catheter during a cooling procedure [25]. However, intravascular catheters can cause bloodstream infections and raise the question about the risk of venous thrombosis. Few cases with thrombosis or thrombophlebitis due to a cooling catheter after a using time of respectively 7 and 10 days have been published [26]. Simosa and colleagues reported in a group of 10 patients with traumatic brain injury that five patients developing a depth venous thrombosis after an average of 5.4 days but concluded that the group under examination already had a high risk for development of thrombosis due to lack of prophylactic anticoagulation [27]. However, the approach towards anticoagulation will be different in survivors after cardiac arrest. The recommendation for the duration of use of the Icy catheter is 4 days (Icy Quattro) but a novel surface coating of the catheter material will soon be approved by authorities to enhance time of use and decrease risk of thrombosis. In addition there might be a higher risk of developing catheter-related bloodstream infections but currently no data are published studying temperature management catheters and infection rates.

### Other cooling methods

The novel RhinoChill intranasal cooling device was able to demonstrate effective reduction of body temperature within the Pre-ROSC Intranasal Cooling Effectiveness trial (PRINCE trial) [28]. The portable system vaporises perfluorchlorcarbon gas with a catheter system into the nasal cavity leading to a fast induction of hypothermia first to the brain as main target organ and second to the body with a slight delay. The intra-arrest cooling approach of the study, starting induction of hypothermia already during CPR, by Castrén and colleagues was conducted as a safety and feasibility study [28]. However, benefit towards survival and neurological outcome was observed in the cooling subgroup, having received CPR within 10 minutes after collapse, although the design of the study was not conceived for outcome analysis. The randomised trial compared in detail prehospital trans-nasal cooling (*n *= 83) with advanced cardiac live support (*n *= 99) and both groups received mild hypothermia on admission to the hospital regardless of the initial rhythm. This method was able to show a significant decrease of tympanic temperature on arrival (34.2°C vs. 35.5°C). Due to the convincing data from the PRINCE trial, the Prehospital Resuscitation Intra Nasal Cooling Effectiveness Survival Study (PRINCESS) started in June 2010 with patient recruitment and is designed to evaluate for good or poor neurological outcome and survival as well as to evaluate the proportion of those achieving ROSC and time to target temperature of 32 to 34°C. First data will be available in June 2013 (ClinicalTrial.gov identifier: NCT01400373). The system has no temperature feedback and the major application area is the induction of hypothermia. Another novel approach is under investigation in the CAMARO trial (ClinicalTrials.gov identifier: NCT01016236).

Following the idea of early and fast induction of hypothermia to improve outcome and decrease side effects after cardiac arrest and incorporate novel data that hypothermia applied before a coronary intervention may reduce the infarct size in STEMI patients, a new automated peritoneal lavage system (Velomedix Inc., Palo Alto, USA) has been developed [22]. The CAMARO trial includes cardiac arrest patients as well as STEMI patients who will be cooled to a target temperature of 34°C without prior resuscitation. The preliminary data of this pilot study, presented as an abstract at the American Heart Association Meeting in Orlando, USA, in November 2011, showed a decrease of temperature to 34°C within 9 minutes, the maintenance phase of 32.5°C was 24 hours in cardiac arrest patients (rewarming 16 hours) and 3 hours maintenance in myocardial infarction patients (rewarming 5 hours). At the moment no device-related complication has occurred with this extremely rapid cooling method [29].

## Discussion

Different cooling methods with varying technical approaches and efficacy are available to deliver mild therapeutic hypothermia to our patients. During cooling the three phases of induction, maintenance and rewarming can be defined. Are different methods necessary to fulfil the requirements in each of these three cooling phases? Taking all mentioned methods together, a combined approach seems to be the optimal way. Particularly with regard to the induction phase a combination of different methods should be suggested to increase the effectiveness of cooling, for example the combination of cold saline and a feedback cooling device, although the optimal overall timing (time to target temperature and cooling rate) is still under debate. In addition to timing, the most important question concerns shivering and its prophylactic successful treatment.

The optimal and most beneficial time point to start hypothermia after cardiac arrest is still not known. The current European resuscitation guidelines recommend starting hypothermia as soon as possible after ROSC. A recently published article by Sendelbach and colleagues revealed the importance of avoiding any time delay of cooling to reach good neurological outcome [30]. This 'earlier is better' strategy can be confirmed by animal data [31-34]. Following the 'earlier is better' strategy, some trials explored the possibility of inducing cooling during resuscitation or directly after ROSC, but data are controversial [35]. Induction of therapeutic hypothermia during prehospital CPR using ice-cold intravenous fluid or intranasal cooling showed that it is feasible and is partially a benefit [28,36]. A major problem in predicting outcome and association with timing and early cooling after cardiac arrest or even during resuscitation with these data is the small sample size and the fact that prehospital hypothermia was discontinued after admission to the hospital in many of these trials [37]. However, the analysis of data from the Scandinavian Hypothermia Network including 986 patients after cardiac arrest by Nielsen and colleagues showed no association of timing towards neurological outcome [38].

Certainly every ICU should provide 4°C cold saline to increase the cooling rate and to reach the target temperature as soon as possible. The administration of cold saline seems a feasible method in the preclinical setting as well as in addition to other preclinical devices available and after admission cold saline can be combined with a feedback device to speed up the cooling. Furthermore, shivering is one of the most important side effects that can occur during hypothermia leading to an increased metabolic rate, high oxygen consumption and heat generation, and therefore needs to be kept in mind to be avoided and treated aggressively. The threshold for this defence mechanism of the thermoregulatory system is around ±35.5°C (1°C below the vasoconstriction threshold) [39,40]. Therefore a fast induction to cross this threshold as quickly as possible seems indicated; additional treatment can include a sufficient analgosedation, magnesium and paralysation, but even the simple method of keeping the hands and feet warm by wearing socks and gloves directly from the beginning of induction of hypothermia can avoid shivering very reliably [41]. In patients with traumatic brain injury undergoing temperature management, the benefit of surface counter warming concerning less shivering and improvement of metabolic profile was reported [42]. However, a high cooling rate during induction with a combination of a feedback-cooling device and several additional conventional cooling methods in combination with hand and feet counter warming as described and a sufficient sedation level seems to be the best way to avoid shivering. In addition, every temperature management procedure requires a reliable core temperature. The gold standard is still the temperature taken directly in the bloodstream (for example, pulmonary catheter) or directly by the cooling device itself as possible with the Philips Accutrol endovascular catheter. Other common places for temperature measurement are the bladder by Foley catheters, oeosophageal probes, tympanic and rectal temperature [43]. Modern temperature management systems with high cooling rates lead to a fast induction of hypothermia that can only be detected by most temperature sensors with a time delay. The closed approach towards the gold standard might be the oesophageal measurement with an approximately average time delay of 5 minutes (range 5 to 10 minutes) [40].

## Conclusion

A wide range of conventional and technical methods exists to apply mild therapeutic hypothermia after cardiac arrest. Hoedemaekers and colleagues compared all described different methods (conventional cold infusion/ice, water blankets, gel-coated pads, intravascular) in ICU patients regarding the speed of cooling (°C/hour) and the reliability to maintain a stable target temperature. The authors conclude that water-circulating blankets, gel-coated pads and intravascular cooling are almost equally efficient for induction but intravascular methods were superior for maintaining the target temperature [44]. Some performance data might have changed over the last years due to the industry having developed the next generation of cooling devices. However, every method has its own partly limited, indication and a combination of an automatic computer-processed feedback device with conventional methods seems a good and safe solution. The type of feedback device used in a hospital (invasive vs. non-invasive) depends on several factors but mainly on the personal preference of the treating doctors, type of patients and the local standard as well. In addition, the way of thinking is changing and it is no longer a question of making the patient cool as good as possible but rather has evolved into a complex temperature management procedure with its own risks and pitfalls as well as benefits for the patient. It is a precondition to ensure a precise and tight temperature control during all three treatment phases. Especially during rewarming, which is a very critical phase of temperature management, close temperature monitoring is necessary and can be easily achieved with a computer-feedback cooling system. A passive, uncontrolled increase of temperature should be avoided in the modern temperature management approach. However, the adoption rate and implementation of hypothermia as part of standard post-arrest care is still not high enough. Reasons are manifold but the latest version of available cooling devices may be able to help to increase the application rate by making the treatment safe and easy. If the hospital team feels confident with the topic of temperature management, numbers of operators might increase, even if the number of cardiac arrest patients treated in a hospital is low.

The presentation of different temperature-management methods and interpretation of their efficiency in the age of daily breaking news about mild hypothermia treatment and widening of the indication can only be a momentary snap-shot and cannot aspire to completeness.

## Competing interests

The author received financial support and material resources from Medivance, Zoll, Philips, EMCOOL and C.R. Bard within different projects and honorarium from Medivance, Zoll and Philips for lectures. This abstract has not been influenced by anyone in collection of data, analysis, interpretation and writing.
